# cncRNAs: Bi-functional RNAs with protein coding and non-coding functions

**DOI:** 10.1016/j.semcdb.2015.10.024

**Published:** 2015-12

**Authors:** Pooja Kumari, Karuna Sampath

**Affiliations:** Division of Biomedical Cell Biology, Warwick Medical School, The University of Warwick, Gibbet Hill Road, Coventry CV47AJ, United Kingdom

**Keywords:** cncRNAs, Bi-functional RNA, Non-coding RNA, Protein-coding, Regulatory RNA, RNA structure, RNA processing, Dual function RNA

## Abstract

For many decades, the major function of mRNA was thought to be to provide protein-coding information embedded in the genome. The advent of high-throughput sequencing has led to the discovery of pervasive transcription of eukaryotic genomes and opened the world of RNA-mediated gene regulation. Many regulatory RNAs have been found to be incapable of protein coding and are hence termed as non-coding RNAs (ncRNAs). However, studies in recent years have shown that several previously annotated non-coding RNAs have the potential to encode proteins, and conversely, some coding RNAs have regulatory functions independent of the protein they encode. Such bi-functional RNAs, with both protein coding and non-coding functions, which we term as ‘*cncRNAs*’, have emerged as new players in cellular systems. Here, we describe the functions of some cncRNAs identified from bacteria to humans. Because the functions of many RNAs across genomes remains unclear, we propose that RNAs be classified as coding, non-coding or both only after careful analysis of their functions.

## 1 Introduction

The ‘one gene one enzyme’ hypothesis proposed by Beadle and Tatum in 1941 [1] and the elucidation of the double helical structure of DNA in 1953 [2] led Crick to propose of the central dogma of molecular biology placing RNA at the center of the directional information flow from genes to their protein products [3]. Subsequent identification of messenger RNAs (mRNAs), adaptor RNA molecules (tRNA) and ribonucleoprotein-dependent catalysis of polypeptide synthesis (rRNA/ribosomes) validated RNA versatility and eventually inspired the first model of RNA-based regulatory networks in cells of higher organisms [4], [5], [6], [7], [8]. However, the discovery of cis-regulatory elements in DNA controlling gene expression by virtue of their interaction with cognate transcription factors captured the imagination and interest of scientists, and for many years, the regulatory roles of RNA were largely ignored.

**Fig. 1:**
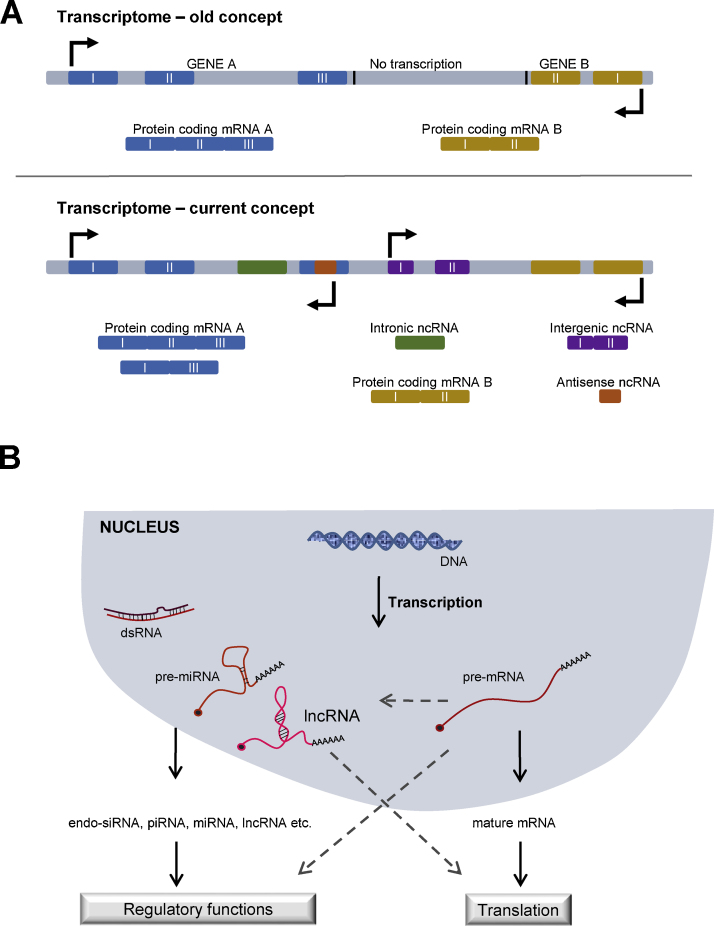
Schematic showing various classes of non-coding RNAs. (A) *Transcription: then and now*. The conventional concept of transcription suggested that only specific loci in the genome are transcribed to code for proteins while current understanding points toward pervasive transcription of the genome and wide spread occurrence of non-coding RNAs. The schematic shows a genomic region with two genes, A (exons in blue) and B (exons in yellow). According to the old concept there is no transcription in the intergenic region between genes A and B. The current concept supports the presence of intergenic ncRNAs (purple, between gene A and B), intronic ncRNAs (green, between exon II and III of gene A) and antisense ncRNA (orange, in opposite orientation in exon III of gene A). Alternative splicing may also lead to different isoforms of RNAs as shown in the second isoform of gene A which lacks exon II. (B) *Crosstalk between coding and non-coding RNAs*. The transcriptome is more complex than anticipated. Protein coding pre-mRNAs can give rise to non-coding RNAs. Long non-coding RNAs can encode for short peptides and protein-coding mRNAs can have additional regulatory functions. (For interpretation of the references to color in this figure legend, the reader is referred to the web version of this article.)

This protein-centric view of gene regulation was challenged by the discovery of small regulatory RNAs (e.g., miRNAs) and gene silencing by RNA interference (RNAi) [9], [10], [11]. Subsequently, the advent of high-throughput sequencing and transcriptome analysis showed that thousands of genomic loci undergo transcription to produce large transcripts that may not code for proteins [12], [13] (Fig. 1A). These findings are supported by the ENCODE (Encyclopedia of DNA Elements) project which showed that ∼80% of the mammalian genome is transcribed [14]. Furthermore, the ratio of non-coding to protein coding transcripts has been proposed to increase with the complexity of organisms and approximately 95% of human transcripts are thought to be non-coding RNAs [15]. The regulatory functions of long non-coding RNAs (lncRNAs) are under active investigation by several groups and have been recently reviewed in [16], [17], [18]. The phenomenal scale of the non-protein coding genome shows that our current understanding of RNA-based gene regulation is rather cursory. Studies in a variety of organisms over the last two decades suggest that RNA molecules contain many more *cis-* and *trans-*regulatory functions than previously thought. Although initially lncRNAs were thought to function strictly as RNAs and not code for proteins, recent studies have showed that many previously annotated non-coding RNAs can recruit ribosomes and encode short peptides [19], [20], [21]. In addition, emerging evidence suggests that even protein coding mRNAs can have structural and/or regulatory functions independent of the protein they encode. [22]. These additional functions of RNA may seem surprising, but are not completely unexpected in light of the view that all current forms of life might have evolved from an RNA world [23], [24]. RNA is a versatile molecule in that RNA can store genetic information similar to DNA, and can also act as a catalyst similar to proteins [25], [26]. In this review, we focus on bi-functional RNAs with both protein coding and non-coding roles (cncRNAs). cncRNAs, carrying both protein coding and RNA-intrinsic functions, call for reviewing the concept where mRNAs were considered a passive step in the transition of genetic information from DNA to protein. These dual function RNAs also present a potential evolutionary link between mRNAs and ncRNAs (miRNA, endo-siRNA, piRNA, lncRNA, etc.), which were previously thought to be inherently different (Fig. 1B). Here, we describe cncRNAs from a variety of organisms ranging from bacteria to humans, with emphasis on the structural or regulatory functions of protein-coding RNAs with roles in development and disease.

## 2 Small regulatory RNAs in bacteria

Small non-coding RNAs have been shown to regulate post-transcriptional gene expression in all kingdoms of life, including bacteria. Bacterial genomes encode a large number of small transcripts (sRNAs) in the range of 50–350 nucleotides. Bacterial sRNAs can be grouped in two classes: (1) antisense RNAs that function via base-pairing with their targets, and (2) protein-binding sRNAs [27]. Most bacterial antisense RNAs are non-coding and are also called ‘ribo-switches’ or ‘ribo-regulators’. However, in recent years it has become evident that some antisense RNAs can also encode peptides [28]. Here, we describe three bi-functional bacterial sRNAs that have been functionally characterized.

### 2.1 RNAIII

*Staphylococcus aureus* RNAIII was the first bacterial sRNA reported to have dual functions. *S. aureus* is a potent pathogen and its virulence is attributed to both cell surface-associated proteins and secreted toxins. The 5′ region of RNAIII encodes a secreted 26 aa peptide, δ-hemolysin (*hld*), which targets host cell membranes, causing lysis [29]. δ-hemolysin does not have any known regulatory functions but RNAIII, a 514 nucleotide long sRNA, regulates stability and translation of virulence factors by direct base pairing with the corresponding transcripts. The expression of cell surface-associated factors is repressed at the end of exponential growth phase while that of secreted factors is stimulated [30], [31]. This reciprocal regulation is carried out by the *agr* locus. RNAIII sRNA is the major effector of the *agr* response [32]. The 3′ region of RNAIII inhibits ribosomal binding and translation initiation of *coagulase* (an enzyme), *staphylococcal protein a* (a cell surface-associated factor), and *rot* (a transcription factor). Consistently, this region of the RNA is more conserved among different isolates of *S. aureus* [33], [34], [35]. The 5′ region of RNA III also functions by base-pairing and facilitates the translation of *α-hemolysin* (*hla*), a secreted factor by preventing the formation of a translational inhibitory complex. This region overlaps with the coding sequence of *hld*, hence the base pairing activity of RNAIII with mRNAs may prevent translation of *hld* [36]. Such a mode of regulation is consistent with a delay in accumulation of δ-hemolysin after RNAIII sRNA synthesis [37]. Hence, it can be envisaged that *hld* is regulated at several levels and there is a possible interplay between production of δ-hemolysin and the antisense functions of RNAIII.

### 2.2 SgrS

**Fig. 2:**
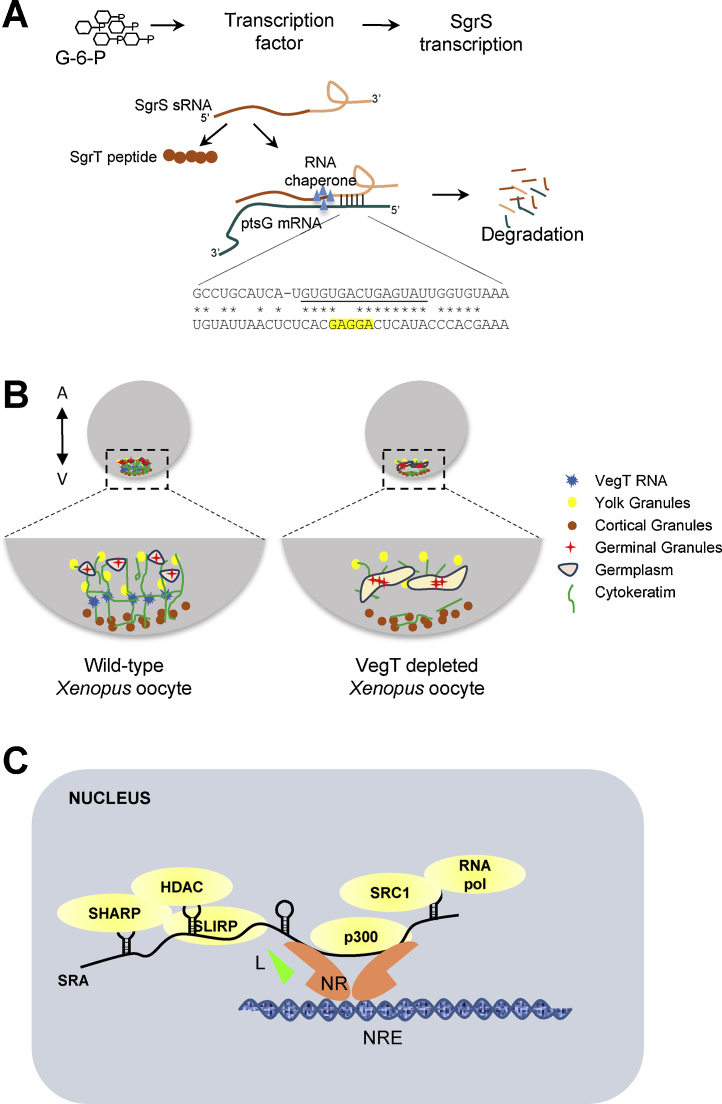
Regulatory functions of coding mRNAs. (A) *Base pairing leads to RNA degradation/translational regulation*. Glucose phosphate stress (G-6-P) leads to activation of transcription of a cncRNA, SgrS. The 5′ region of SgrS encodes for a short peptide (SgrT) while the 3′ region regulates the expression of ptsG mRNA by base pairing. The minimal base pairing region of SgrS RNA is underlined and the Shine–Dalagarno (SD) sequence of ptsG mRNA is highlighted. This base pairing leads to translational repression and RNA degradation. (B) *Structural role of RNA in cytoskeletal organization.* Here a *Xenopus* egg is depicted along the animal (A) – vegetal (V) axis and the vegetal cortex (boxed) is illustrated in detail. In *Xenopus* oocytes, cytokeratin (green filaments) form a complex interconnected network spanning between the cortical granules (brown) and the yolk granules (yellow) at the vegetal cortex. Germplasm islands (pink) are anchored at the vegetal pole by the cytokeratin network. Germinal granules (red) are located within these islands. Proper organization of cytokeratin network requires VegT RNA (blue). In VegT depleted oocytes, long cytokeratin filaments are disintegrated, and the fragmented cytokeratin network affects germplasm distribution such that the islands and individual germinal granules fuse into larger aggregates. (C) *RNA as a scaffold to assemble regulatory complexes*. Several co-regulators participate in nuclear receptor signaling. In absence of ligand (L), repressors such as SHARP and SLIRP bind the nuclear receptors (NR) and repress transcription by mobilizing histone deacytylases (HDAC). Upon ligand binding, the repressors are replaced by co-activators (e.g., SRC-1 and p300), that in turn recruit RNA polymerase II and initiate target gene expression. SRA, the RNA co-regulator is thought to function as a scaffold and brings the whole complex together at the nuclear response element (NRE) and facilitates gene regulation. (For interpretation of the references to color in this figure legend, the reader is referred to the web version of this article.)

SgrS (Sugar transport related), a 227-nucleotide sRNA, is induced during glucose-phosphate stress conditions resulting from disruption of glycolytic flux and accumulation of glucose-6-phosphate. SgrS actively alleviates stress by negatively regulating the stability and translation of the major glucose transporters, *ptsG* and *manXYZ*, via base pairing [38], [39]. In addition to this base-pairing antisense activity, SgrS codes for a 43-amino acid peptide, SgrT [40]. Interestingly, SgrT also functions in the glucose–phosphate stress response, but by unrelated mechanisms. Ectopic expression of SgrT from constructs lacking the base-pairing sequences eases the stress response while the stability of transporters is not affected. It has been suggested that SgrT functions by inhibiting the active components of glucose transported but the precise mechanisms are not clearly understood [40]. Vanderpool and colleagues identified a highly conserved 15-nucleotide sequence at the 3′ end of SgrS from several enteric species, even though the overall sequence was rather divergent [41]. These conserved nucleotides are complementary to the ribosomal binding site in *ptsG* mRNA, and hence base pairing leads to translation repression and mRNA degradation (Fig. 2A) [42]. In case of *manXYZ* polycistronic mRNAs, SgrS base pairs with the coding sequence of *manX* leading to RNA degradation [38], [39]. In contrast to RNAIII, the regulatory sequences and coding sequences are spatially separated in SgrS. The coupled degradation of SgrS during ribo-regulation suggests that the two functions of SgrS are mutually exclusive and the same RNA molecule cannot serve as both ribo-regulator and a template for translation of SgrT. Further investigation is required to study if there is any relationship between the regulatory function and translation of SgrS.

### 2.3 SR1

SR1 is a dual function RNA identified in the gram-positive bacterium, *Bacillus subtilis.* SR1 represses the translation of a transcriptional activator *ahrC* that regulates the arginine catabolic operons, rhoABC and rhoDEF [43]. There are seven regions of complementarity between SR1 and ahrC. SR1 binding inhibits translation of ahrC mRNA by inducing structural changes downstream of the ribosomal binding site [44]. In a quest to discover more targets of SR1, Brantl and colleagues discovered that SR1 also regulates the glycolytic *gapA* operon [45]. However, the mechanism of SR1-mediated regulation of *gapA* is not by base pairing of the RNA. SR1 encodes a 39-aa peptide (SR1P) that stabilizes gapA operon RNA. SR1P was reported to directly bind to GapA protein, but the mechanisms underlying this mode of regulation are not fully understood [45].

## 3 Bi-functional RNAs in plants

Plants exhibit a remarkable developmental plasticity and extensively regulate their gene expression profiles in response to environmental cues and stress. RNA-mediated regulation appears to play a significant role in adaptation to varying environmental conditions [46]. Here, we discuss two plant RNAs that code for peptides and also have intrinsic function as RNAs.

### 3.1 ENOD40

*Early nodulin 40* (*ENOD40*) is the best-studied example of a cncRNA in plants. It was first identified as a gene expressed during early stages of root nodule formation, resulting from the symbiotic association of leguminous plants with rhizobial bacteria [47]. ENOD40 is expressed in differentiating cells of nodule primordia and the expression levels of *ENOD40* positively correlate with the rate of nodulation in transgenic plants [48]. Due to the absence of any long open reading frame (ORF) and the highly stable secondary structure of the RNA, *ENOD40* was proposed to be a non-coding RNA. However, the molecular mechanisms underlying its activity remained unclear for many years [49], [50]. Later, studies in *Medicago truncatula* (a model legume plant) showed that there are two conserved short ORFs in the *ENOD40* transcript and that the 5′ORF is highly conserved [51]. Transient expression of *ENOD40* in roots resulted in cortical cell divisions at high frequency. By targeting wild type and truncated/mutated *ENOD40* to the cortical cells in roots, Crespi and colleagues showed that translation of both short ORFs is required for the activity of *ENOD40.* Interestingly, deletion of an inter-ORF region of the RNA with a predicted secondary structure also affected the activity of *ENOD40*, without altering translation of the ORFs. These results emphasized the importance of both the RNA structure and short ORFs, and imply a dual role for *ENOD40* RNA in plant roots.

Yeast-three-hybrid studies showed that a novel protein, MtRBP1, interacts with *ENOD40* RNA. MtRBP1 was found to be cytoplasmic in nodule primordia cells expressing high levels of *ENOD40*, whereas MtRBP1 localized to nuclear speckles in other root cells. Consistent with this, upon expression of *ENOD40* in heterologous cells, MtRBP1 relocated from the nucleus to the cytoplasm. While the short ORFs encoded by *ENOD40* did not play a role in localization of MtRBP1, the RNA was found to be directly required for cytoplasmic localization of MtRBP1. The function of this RNP (ribo-nucleo protein) is still unknown, although it has been proposed to function as a translational regulator in the cytoplasm [52]. Furthermore, in soybean, the two short peptides encoded by *ENOD40* bind to sucrose synthase (SUC1) and inhibit phosphorylation. Phosphorylated SUC1 undergoes proteasomal degradation. Thus, ENOD40 peptides regulate the turnover of SUC1. These diverse functions substantiate the bi-functional nature of *ENOD40*. Recent studies in *Arabidopsis* and rice have identified a number of RNAs similar to *ENOD40* that can code for short ORFs [53], [54]. It is conceivable that these are also potential cncRNAs.

### 3.2 MtHAP2-1

It has been observed that short ORFs in the 5′UTR (upstream ORF, uORF) of an RNA can contribute to gene regulation [55]. For instance, a HAP2 family transcription factor in *M. truncatula*, MtHAP2-1, is regulated by a peptide, uORF1p, which is encoded by its uORF. MtHAP2-1 is a key regulator in the nodule meristem and functions in nodule development. Alternative splicing of the first intron in the 5′UTR of MtHAP2 is predominant during nodulation, and results in production of uORF1p. Unlike other uORFs that regulate translation by ribosomal stalling, uORF1p represses translation by binding to the 5′UTR of MtHAP2-1 [56]. This regulation is important for spatial regulation of MtHAP2-1 and nodulation. Hence, MtHAP2 is an example of a cncRNA whose alternative splicing results in dual functions of the RNA.

### 3.3 miRNA-encoded peptides (miPEPs)

A recent report from Combier and colleagues shows that some pre-miRNA transcripts in plants have functional ORFs [57]. The highly conserved pre-miRNA sequence of *M. truncatula* miR171b with only 0.85% SNPs, suggested the possibility of ORFs in the sequence. Indeed, two ORFs were found in the 5′ region of pre-miRNA 171b, encoding short peptides of 5 and 20 amino acid residues, respectively. Further analysis with a β-glucuronidase (GUS) reporter showed that only the ORF encoding 20-amino acid peptide named as miPEP171b is expressed and translated at the lateral root initiation site. Interestingly, miPEP171b specifically enhances the expression of miR171b, and not other miRNAs when overexpressed as pre-miRNA in *M. truncatula* roots and in tobacco leaves. Addition of synthetic miPEP171b to the seedlings of *M. truncatula* increased the levels of miR171b and affected lateral root development. Analysis of 50 pre-miRNA sequences from *Arabidopsis thaliana* showed presence of at least one ORF in each sequence [58]. Interestingly, overexpression of various miPEPs encoded by pre-miRNA of different classed in *M. truncatula* and *A. thaliana*, positively correlated with accumulation of corresponding miRNAs. Inhibition of RNA synthesis during overexpression of miPEPs and analysis in RNA polymerase subunit mutants suggest that miPEPs function as transcriptional regulators of the corresponding miRNAs [57]. Further studies are required to understand how cytoplasmic translation of pre-miRNA and nuclear maturation of miRNAs is regulated. The discovery of miPEPs further strengthens the concept of bi-functional cncRNAs, and it will be interesting to determine if miPEPs exist in other organisms.

## 4 Bi-functional RNAs in animal development

Early embryogenesis of many animals relies on a large number of transcripts maternally deposited in the oocytes and mediating first steps of development prior to commencement of the zygotic gene expression program. Some of these maternal RNAs are required for oocyte maturation while others are stored in the form of mRNPs and are translated and/or degraded in an orchestrated manner during early phases of embryonic development. Hence, maternally deposited RNAs are under tight post-transcriptional regulation that includes regulated processing, localization and translation [59], [60], [61], [62], [63]. It is widely believed that the major biological function of localization and translational control of RNAs in oocytes and embryos is spatial and temporal regulation of the corresponding protein product. However, studies in *Xenopus*, *Drosophila* and more recently in zebrafish suggest that besides coding for proteins, localized RNAs can have additional non-coding functions.

### 4.1 *Xenopus* VegT

*VegT* was identified as a maternal RNA localized to the vegetal cortex *Xenopus laevis* oocytes. VegT codes for a T-box transcription factor that patterns the mesendoderm along the dorso-ventral axis [64]. Heasman et al. first reported that depletion of VegT mRNA leads to disruption of vegetal localization of maternal mRNAs such as Vg1 [65]. Following this, Kloc and colleagues discovered that VegT mRNA and a non-coding RNA Xlsirts, play structural roles in the organization of the cytoskeleton at the vegetal cortex of *Xenopus* oocytes, and that the vegetal cytoskeleton is important for anchorage of germ-line specific RNAs and formation of the germinal granules [66]. Depletion of either VegT or Xlsirts RNA by injection of antisense oligonucleotides specifically disrupted the cytokeratin network at the vegetal cortex. However, translation-blocking antisense morpholinos against VegT mRNA did not affect cytokeratin structure. Additionally, upon injection of synthetic VegT mRNA into the VegT depleted oocytes, the cytokeratin structure was restored. These lines of evidences suggested that *VegT* has an mRNA-intrinsic function. [66], [67]. Further studies by Kloc and colleagues to analyze the three dimensional ultra-structure of cytoskeleton showed that *VegT* mRNA molecules are integrated into the multilayered cytoskeleton which collapses and disintegrates in the absence of RNA. The integrity of the cytoskeleton is important for correct distribution of the germ plasm and germinal granules at the vegetal cortex (Fig. 2B). Based on these findings, *VegT* mRNA has been suggested to have a structural function in germ-line development, independent of the function of VegT protein in germ layer patterning [67].

### 4.2 *Drosophila* oskar

*Oskar* (*osk*) was identified as a maternal-effect gene required for antero–posterior patterning during *Drosophila* embryogenesis [68]. During early oogenesis, osk mRNA is transported from nurse cells to the developing oocyte. Subsequently, osk mRNA is actively transported to the posterior pole, where Osk protein is exclusively synthesized from localized osk RNA. Prior to localization, osk mRNA is translationally repressed by Cup, a 4E binding protein. Cup regulates osk mRNA by interacting with an RNA-binding protein, Bruno, which recognizes specific sequence motifs in osk mRNA. Cup competes with eIF4G for binding to eIF4E, the protein that binds to the 7-methyl-guanosine cap structure in mRNAs. Interactions between eIF4G and eIF4E are required for ribosomes to load on mRNAs, so sequestration of eIF4E by Cup blocks translation [69]. Posterior localization and localized translation of osk mRNA determines the site for formation of primordial germ cells and the abdomen. Osk protein is known to regulate its own RNA localization and functions as a scaffold for the assembly of the germ plasm [70], [71]. The classical *osk* mutants identified in the maternal effect screen that produced embryos lacking abdomen and germ cells lacked functional Osk protein but still expressed mRNA [72]. Surprisingly, two new *osk* alleles with reduced or no osk mRNA showed more severe and earlier defects during oogenesis compared to *osk* alleles that express mRNA. Females harboring RNA null mutations failed to lay eggs and were sterile as a result of an early arrest during oogenesis [73]. The oogenesis arrest was complemented by nonsense mutant alleles which still expressed osk mRNA, suggesting that the early oogenesis function of *osk* is mediated by osk RNA and not Osk protein. To confirm this possibility, in a series of elegant experiments, Ephrussi and colleagues showed that overexpression of merely the osk 3′UTR was sufficient to rescue the eggless phenotype of osk RNA-null mutants. Therefore, they suggested that the osk 3′UTR might function as a scaffold to assemble RNP complexes that are required for oocyte development [73]. In agreement, a recent study shows that loss of oskar RNA leads to accumulation of germline regulatory factors in the somatic follicle cells and specific elements in the oskar 3′UTR sequester the translation regulator, Bruno in the oocyte [74]. Taken together, these studies show that osk functions as a protein coding-mRNA during embryogenesis and a non-coding RNA during early oogenesis, and hence qualifies as a cncRNA. The molecular mechanisms underlying the non-coding function of osk RNA are just beginning to be understood.

### 4.3 Zebrafish squint

Squint (Sqt) is a Nodal-related signaling molecule belonging to the transforming growth factor beta (TGFβ) superfamily. Nodal signaling plays important roles during embryonic development with essential functions in germ layer patterning [75], [76]. The role of Nodal signaling in mesendoderm induction and patterning, specification of the ventral neural tube, and left–right axis specification has been well studied [75], [77], [78], [79], [80], [81]. In addition to these known roles, we discovered a novel non-coding function of asymmetrically localized maternal sqt/nodal transcripts in dorsal axis specification [82], [83]. In mature oocytes, sqt transcripts are distributed uniformly throughout the yolk, and form discrete puncta upon egg activation and fertilization. Subsequently, these sqt RNA puncta form bigger aggregates and translocate to the blastoderm by a microtubule-dependent mechanism [84]. By the 4-cell stage, sqt RNA is asymmetrically localized to one or two cells and the cells acquiring sqt RNA are required for the formation of dorsal structures [82]. Removal of sqt-containing cells or depletion of maternal sqt by anti-sense oligonucleotides resulted in embryos with severe deficiencies in embryonic dorsal structures. These experiments suggested that asymmetrically localized sqt RNA may function in dorsal axis specification. However, embryos obtained from homozygous insertion mutants affecting *sqt* exhibit mild dorsal defects, raising questions regarding the contribution of maternal sqt in dorsal specification [85], [86]. Interestingly, we observed that while the insertion mutants for *sqt* do not make functional protein, mutant sqt RNA is expressed and localized in homozygous *sqt* insertion mutant embryos. Furthermore, mutant sqt transcripts expand dorsal progenitors in early zebrafish embryos. Using a variety of mutations that disrupt Sqt protein, we showed that sqt RNA functions in the initiation of embryonic dorsal, independent of Sqt protein. Over-expression of the sqt 3′UTR sequences rescues the dorsal defects resulting from depletion of maternal sqt. Subsequent analysis of sqt RNA function in maternal mutants affecting Wnt and Nodal signaling showed that the dorsalizing function of the sqt 3′UTR requires Wnt/β catenin signaling [83], but Nodal signaling per se is not required for initiation of dorsal specification. These findings are consistent with the requirement of Nodal receptors and the Nodal co-receptor, One-eyed pinhead (Oep), from late blastula stages [87], [88]. Based on these results we proposed a role for maternal sqt RNA in binding and transporting factor(s) via its 3′UTR, to the future dorsal side during early blastula stages prior to the signaling functions of Sqt protein. Such a binding factor (or complex) is likely to function via the canonical Wnt/β catenin pathway. Identification of the factors that bind to sqt 3′UTR can provide insights into the mechanisms by which sqt RNA and particularly the 3′UTR controls dorsal axis formation via Wnt signaling.

**Fig. 3:**
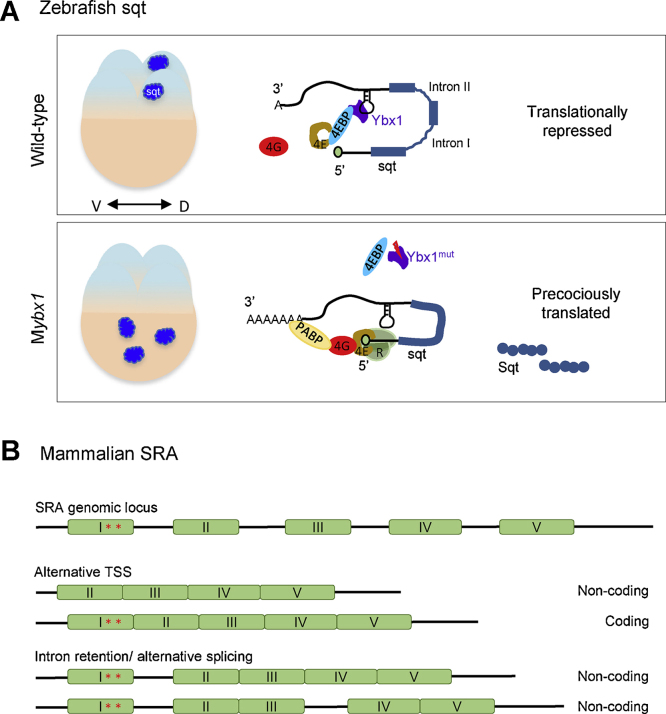
RNA processing facilitates dual function of cncRNAs. (A) Regulated splicing, polyadenylation, and translation in temporal partitioning of non-coding versus coding functions of sqt RNA in zebrafish. A 4-cell stage zebrafish embryo is depicted with dorsal progenitor cells (D) at the right side. In wild-type embryos, by the 4-cell stage, sqt transcripts are actively localized to 1 or 2 cells. In the schematic representation of sqt RNA, black lines represent UTRs, blue boxes represent the 3 coding exons and the blue lines represent the introns. Maternal RNA is not completely spliced, lacks a poly A tail and is translationally repressed by Ybx1. Ybx1 sequesters eIF4E (4E) either directly or in a complex with an eIF4E binding protein (4EBP) to prevent formation of the eIF4 translation pre-initiation complex and recruitment of ribosomes (R). In maternal *ybx1* mutant (M*ybx1*) embryos, sqt RNA fails to localize and forms aggregates in the yolk. Maternal sqt RNA is precociously spliced, polyadenylated, and Sqt protein is translated prematurely in M*ybx1* mutant embryos. This leads to premature activation of the Nodal/Squint pathway in M*ybx1* mutants. (B) Alternative transcription start sites, intron retention and alternative splicing result coding and non-coding isoforms of SRA RNA. The SRA genomic locus consists of five coding exons, and exon I has two in-frame start codons (red asterisks). There is an alternative transcriptional start site (TSS) in exon I which leads to the production of a non-coding isoform of SRA. Alternative splicing leads to retention of intron I and sometimes intron III, and can also produce non-coding SRA isoforms. (For interpretation of the references to color in this figure legend, the reader is referred to the web version of this article.)

We also uncovered another level of regulation that likely controls the coding and non-coding functions of sqt in a spatial and temporal manner. Consistent with the non-coding function of sqt RNA in early embryos, maternal sqt RNA is translationally repressed during early cleavage stages [89]. Y-box binding protein 1 (Ybx1), a conserved nucleic acid binding protein, is required for dorsal localization of sqt and translational control of Sqt/Nodal signaling in early zebrafish embryos. Ybx1 binds to a localization element in the sqt 3′UTR, and to cap-binding protein eIF4E, and prevents Sqt protein translation in early embryos. Maternal sqt RNA is deposited in an unprocessed form in the egg, i.e., it is un-spliced and non-polyadenylated [83], [90]. The RNA gets completely processed only by the 16-cell stage. In contrast, spliced and polyadenylated sqt was detected in embryos obtained from homozygous *ybx1* females as early as the one-cell stage, indicating premature processing of the mRNA in mutant embryos. Consistent with this observation, Sqt protein is precociously translated in maternal *ybx1* mutants compared to wild-type embryos. This leads to premature and deregulated Squint/Nodal signaling, which is catastrophic for embryonic development and maternal *ybx1* mutants typically do not survive beyond early gastrula stages [89]. Thus, sqt mRNA presents an example of a cncRNA where RNA processing and translation regulate the coding and non-coding functions of the RNA, such that they are temporally distinct events during embryonic development (Fig. 3A).

## 5 Epigenetic regulation by RNAs

RNA molecules actively participate in epigenetic regulation by physically interacting with chromatin modifying enzymes. They are involved in modification of histones and DNA methylation [91]. Studies so far suggest that RNA-mediated epigenetic regulation is carried out for the most part by nuclear lncRNAs and lncRNAs have been shown to function as both activators (*HOTTIP*, *Mistral*, etc.) and repressors (*HOTAIR*, *ANRIL*, *Xist*, etc.) (reviewed in [92], [93]). However, recent data from Coolen and Esteller laboratories suggests that coding RNAs may also be involved in epigenetic regulation [94]. By specifically looking for RNAs that are bound to SUZ12, a component of the polycomb repressive complex 2 (PRC2), in chemically cross-linked samples of human prostate cancer cell lines, Coolen and colleagues identified a number of protein coding RNAs that bind to SUZ12 with affinities comparable to that of lncRNAs [94]. They also re-analyzed a similar data set from the Esteller lab where RNAs bound to EZH2, another component of PRC2, were immune-precipitated and sequenced, and identified protein-coding RNAs [95]. This study was performed in human colorectal cancer cell lines. Analysis of RNA-sequencing data obtained from mouse embryonic stem cells also identified protein-coding RNAs that bind to EZH2 [96]. Although the functional significance of such PRC2-mRNA interaction are yet to be discovered, taken together these studies suggest that even protein-coding RNAs can participate in epigenetic regulation.

## 6 Bi-functional RNAs in disease

Mutations leading to dysfunctional RNAs can lead to a variety of human diseases ranging from neuro-degeneration to cancer. Here, we describe the pathological function of some RNAs independent of their protein function.

### 6.1 SRA, a dual function co-regulator of transcription factors

Steroid receptor RNA Activator (*SRA*) was the first mammalian RNA to be discovered with dual roles, protein coding and non-coding, in myogenic differentiation [97]. SRA was initially identified as a partner of progesterone receptor with co-regulatory functions [98]. Despite the presence of a long ORF, Lanz and colleagues did not detect a protein product encoded by SRA mRNA. They then tested the ability of SRA to co-activate glucocorticoid receptor in the presence of a de novo protein synthesis inhibitor, cyclohexamide and concluded that SRA functions as an RNA co-activator of nuclear receptors [98]. Soon thereafter, a number of studies demonstrated that SRA co-activates many nuclear receptors including the estrogen, androgen, gluco-corticoid and retinoic acid receptors. Secondary structure prediction of SRA RNA followed by mutational analysis suggested the presence of multiple stem loops in SRA RNA that are required for its activity [99]. SRA functions as a scaffold that brings together transcriptional co-activators, RNA polymerase as well as gene insulators/repressors (reviewed in [100], [101], [102]) (Fig. 2C). The activity of transcription factors such as MyoD and GATA-3 is also enhanced by SRA RNA [103], [104]. Subsequent sequence analysis to identify the transcription start site showed the presence of a novel isoform of SRA, containing an additional 5′ exon. The 5′ exon contains two ATG start codons in the same frame that could potentially lead to the translation of either a 224 or a 236-aa SRA peptide (SRAP). The authors confirmed the presence of the coding RNA isoform and doublet of corresponding peptides by reverse transcription and western blot analysis respectively [97]. Differential transcriptional start site and alternative splicing, resulting in either retention or exclusion of the first and sometimes third intron, determines whether *SRA* functions as a coding or a non-coding RNA (Fig. 3B) [105], [106]. SRAP is conserved among chordates and one of the domains found in all annotated SRAPs contains a RNA recognition motif (RRM), a putative nuclear localization signal and a motif that might interact with nuclear receptors [107]. Using silent mutations that disrupted regulatory motifs in SRA RNA and nonsense mutations that disrupted SRAP, it was established that SRAP functions in both activator and repressor complexes of nuclear receptors, independent of SRA RNA [108], [109], [110]. In contrast, muscle differentiation studies showed that SRAP prevented SRA RNA-dependent activation of MyoD [103].

Interestingly, SRA RNA is expressed at higher levels in human breast tumors as compared to adjacent tissues, and the levels increase with tumor progression [111], [112]. SRA ncRNA and SRAP co-exist in breast cancer cell lines and the relative expression of the two molecules differs in different phenotypes, with higher levels of non-coding SRA detected in invasive cell lines. This suggests that the balance between the two isoforms might define tumor phenotypes and alter gene expression during tumor progression [113]. These data also highlight the role of alternative splicing in tumor metastasis. SRAP is known to function as a co-activator of androgen receptors in prostate cancer. However, its precise role in tumor progression in this context is not fully understood, and it is unknown if non-coding SRA RNA has a role in prostrate tumors [108], [114].

Taken together these studies show that the *SRA* locus codes for various SRA RNA isoforms that have either coding or non-coding functions, and that in some contexts, the coding and non-coding functions can be intertwined. Importantly, the balance of SRA isoforms is relevant to both normal differentiation and disease.

### 6.2 DMPK in myotonic dystrophy

**Fig. 4:**
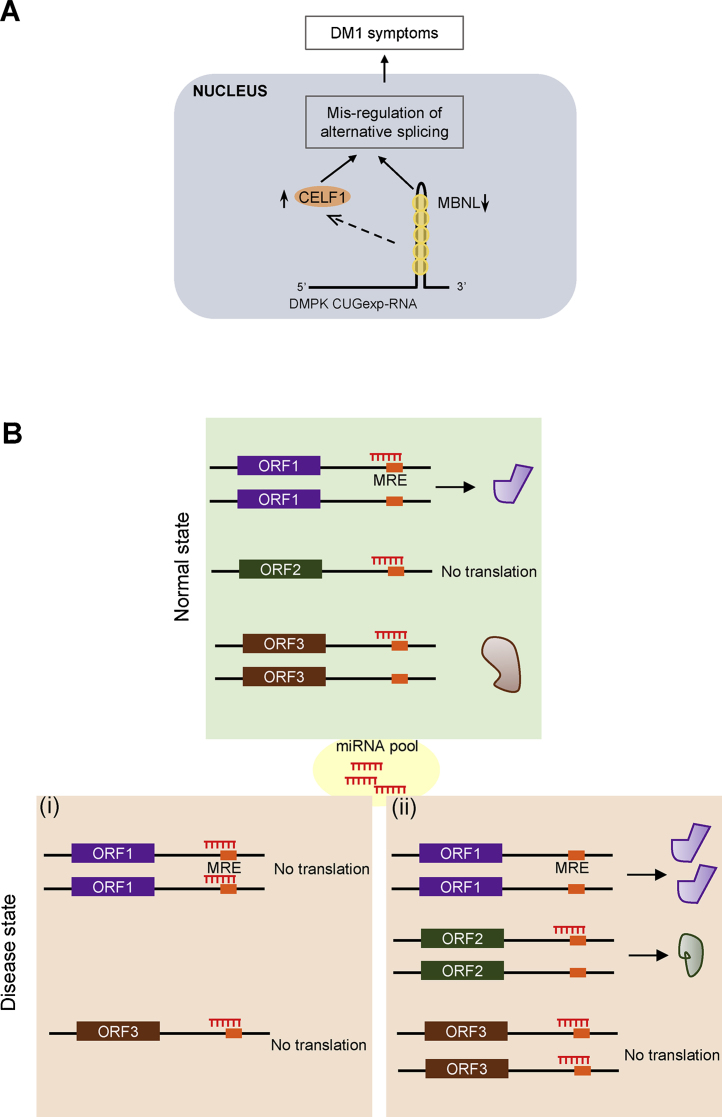
cncRNAs in disease. (A) *Sequestration and modulation of regulatory proteins by DMPK mutant RNA in type I myotonic dystrophy*. Variable length polymorphism resulting from increased number of CUG repeats in 3′UTR of DMPK gene leads to transcription of a toxic form of RNA (DMPK CUGexp-RNA). The CUG repeats form a stable stem loop (the hairpin in the DMPK CUGexp-RNA). The splicing factor MNBL (yellow) directly binds to the CUG repeats. Sequestration of MBNL makes it inactive. DMPK CUG-exp RNA stabilizes another splicing factor, CELF1 by indirect hyperphosphorylation. This leads to mis-regulation of alternative splicing and manifestation of type I myotonic dystrophy (DMI) symptoms. (B) *ceRNAs as miRNA sponge*. A group of mRNAs sharing a particular miRNA response element (MRE) function as ceRNAs and influence each other's translation. In a normal state, a limited pool of miRNAs can regulate the translation of a number of mRNAs in a ceRNA network. When the expression of one mRNA is changed, the redistribution of available miRNA molecules will result in a change in translational output of other mRNAs in the network, potentially leading to disease states. In this schematic, ORF2 functions as a miRNA sponge to regulate the translational output of ORF1 and ORF3. When ORF2 expression is reduced (disease state i) miRNA that was bound to ORF2 will be available to target other RNAs leading to repression of ORF1 and ORF3 while when ORF2 is overexpressed (disease state ii), translational repression of ORF1 and ORF3 by miRNA will be alleviated due to presence of more MREs. (For interpretation of the references to color in this figure legend, the reader is referred to the web version of this article.)

Myotonic dystrophy (DM) is an autosomal dominant inherited disease characterized by slow progressing multi-systemic symptoms like muscle wasting, myotonia, cardiac defects and reduced cognitive ability. By positional cloning, the DM1 mutation associated with type 1 myotonic dystrophy was identified as a variable length polymorphism which resulted from increased number of trinucleotide CUG repeats in the 3′UTR of DM protein kinase (*DMPK*) expressed in tissues affected by myotonic dystrophy [115], [116]. The severity of clinical symptoms of myotonic dystrophy correlates with the number of CTG repeats found in patients [117], [118]. Unaffected individuals have less than 38 repeats whereas patients have between 50 and 1500 repeats. Mutant *DMPK* mRNA with expanded CUG repeats (CUGexp-RNAs) is transcribed but the transcripts are sequestered as discrete foci in nuclei leading to cytoplasmic depletion of *DMPK* mRNA [119]. Haplo-deficiency of DMPK protein and/or SIX5 encoded by the downstream gene leads to delayed onset of mild symptoms, but were not found to be completely responsible for DM1 phenotypes [120], [121]. However, recent evidence suggests that the DM1 pathology involves a toxic gain of function by mutant CUGexp-RNA. Structural and biochemical experiments showed that the CUG repeats form a stable hairpin structure [122], [123]. Moreover, CUGexp-RNA is not transported to the cytoplasm and forms discrete aggregates at the periphery of nuclear speckles, which are structures enriched with splicing related factors [124]. The hairpin structure sequesters developmentally regulated splicing factors like MBNL (Muscle blind like) [125]. Another splicing factor, CELF1 (CUGBP1) also bind to single stranded CUG sequences but do not co-localize with the nuclear aggregates of CUGexp-RNAs. However, expression of CUGexp-RNA leads to hyper-phosphorylation and stabilization of CELF1 [126], [127]. Mis-regulation of MBNL and CELF1 disrupts splicing of a subset of RNAs and lead to embryonic splicing patterns in adult tissue, and hence has a primary role in development of myotonic dystrophy [128]. Thus, repeat expansion of certain nucleotides can convert an mRNA into a functional RNA implicated in protein sequestration and human disease (Fig. 4A). Expansion of similar triplets (CGG, GAA), which are capable of base pairing, in other RNAs have been found associated with a number of other human diseases such as fragile X tremor ataxia syndrome and Friedreich ataxia [129].

### 6.3 p53 RNA in mammalian breast cancer cells

Disruption of *p53*, a critical tumor suppressor gene, is the most frequent single gene event leading to human cancers. The p53 protein is post-translationally modified and rendered active as a transcription factor in response to stresses such as DNA-damage, hypoxia, nutrient deprivation and telomere damage which can lead to cancer. Activated p53 initiates a program of cell cycle arrest and apoptosis [130], [131]. The p53 protein is expressed as at-least four different isoforms resulting from alternative initiation codons. These isoforms were found differentially expressed in human breast cancer samples as compared to normal breast tissue [132], [133]. Mdm2, an E3 ubiquitin ligase is a major regulator of p53 protein and prevents excessive and persistent p53 activation via a feedback regulation [134], [135]. Recently, it was shown that there is an additional feed forward regulation, wherein p53 mRNA interacts with Mdm2 and leads to enhanced p53 translation and stabilization [136]. Mdm2 associates with p53 polysomes via its RING domain and probably enhances translation. Consistent with this possibility, a CUA to CUG mutation in p53 was identified in a lymphocytic leukemia patient where mutant p53 was found to impair Mdm2-mediated enhancement of p53 translation [137]. p53 mRNA recruits Mdm2 to p53 polysomes where the latter likely functions as a chaperone for p53 protein folding. During this process, the E3 ligase activity of Mdm2 is inhibited [136], [138]. Thus, p53 mRNA acts as a switch that controls Mdm2 regulation of p53 protein. Interestingly, the region of p53 mRNA that encodes for the Mdm2 binding site in p53 protein, also interacts with the RING domain of Mdm-2 [136]. This is an example where the same region of RNA mediates both coding and non-coding functions. Mutations in such RNAs should be designed carefully because they have the potential to affect both the activity of the RNA and the encoded protein and lead to binary phenotypes.

### 6.4 Competing endogenous RNAs in cancer

Coding and non-coding transcripts can function as a sponge to bind miRNAs and alleviate the repressive activity of miRNAs on the target mRNAs. Such RNAs that regulate the activity of other RNAs by directly competing for miRNA binding are named as competing endogenous RNAs (ceRNAs) and any perturbation in their levels can lead to disease states [139] (Fig. 4B). One of the best-studied examples of ceRNA regulatory networks is one encompassing the tumor suppressor gene *PTEN*. PTEN encodes a phosphatase that antagonizes the highly oncogenic PI3K/Akt signaling pathway. Various co-expressed RNAs like VAPA, CNOT6L and PTENP1 (a non-coding pseudogene of PTEN) were found to share miRNA response elements (MREs) with PTEN. Their RNAs were shown to relieve miRNA-mediated repression of PTEN. Consistently, Pandolfi and colleagues showed that copy number loss of these ceRNAs during cancer promotes tumorigenesis by repressing PTEN. These interactions were shown to be reciprocal as PTEN mRNA can also regulate the expression of VAPA protein [140]. Thus, the ceRNAs exhibit a regulatory function in addition to their protein coding function. Many such RNAs that function in a miRNA dependent cross-talk and their regulatory functions in tumor suppression have been identified (reviewed in [141], [142]).

## 7 Conclusion and perspectives

**Fig. 5:**
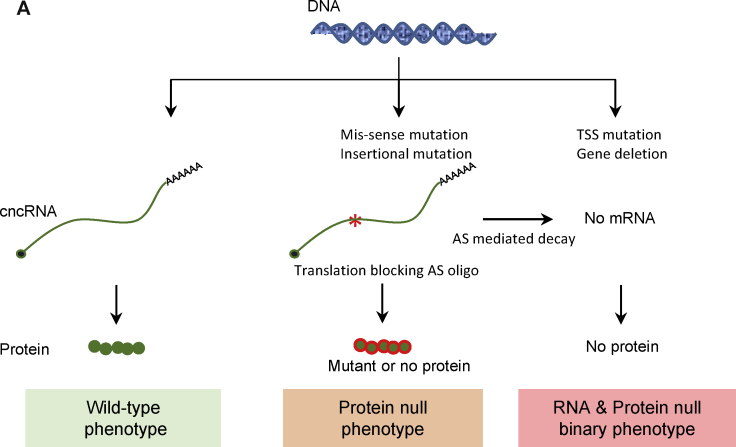
Binary phenotypes – protein null versus RNA null. Normal transcription and translation of a cncRNA will result in wild-type phenotype. Mis-sense or insertion mutations in the genome can result in mutant RNA (red asterisk) that might be stable if not targeted by non-sense mediated decay pathway, and can carry out the non-coding function. So, a protein mutant phenotype will be observed without affecting the activity of the RNA. But mutations that eliminate the transcript (transcription start site or TSS mutations and gene deletions) or antisense oligos that degrade RNA will lead to a binary phenotype resulting from loss of both RNA and protein function. (For interpretation of the references to color in this figure legend, the reader is referred to the web version of this article.)

Here, we reviewed the emerging class of bi-functional RNAs that combine protein-coding and noncoding functions in a single RNA molecule. The current list of these molecules might be limited, but phylogenetic analysis and RNA structure predictions suggest that this list is likely to expand in the future [143], [144]. Indeed, a large number of ncRNAs lacking canonical ORFs are transcribed by polymerase II, spliced, capped and polyadenylated just like mRNAs [145], [146]. It remains an open question what fraction of these can be translated into short functional polypeptides. On the other hand, the protein-centric view that has dominated molecular biology since its inception might have biased characterization of mRNAs to their ‘information messenger’ role leaving a wealth of structural and/or regulatory functions largely unexplored. An important future challenge will be to understand how these cncRNAs balance their coding versus non-coding capacities. Do they partition the two functions to physically distinct domains (as exemplified by bacterial SgrS, *Drosophila* osk and zebrafish sqt) or alternatively, do they utilize the primary sequence for encoding proteins while reserving the secondary or tertiary structure of the same region for non-coding roles? Determining cncRNA conformation using emerging experimental approaches [147], [148] should improve our understanding of how these varied functions are elicited. In addition, for many of the known cncRNAs such as SRA RNA, regulated processing events such as alternative splicing, cleavage and polyadenylation underlie their ability to perform coding versus non-coding functions. A recent study suggested that about 300 alternatively spliced bi-functional RNAs might exist in the human genome [149]. Therefore, we propose that the loss of RNA function phenotypes be examined for identifying new cncRNA loci, as protein-null phenotypes might be distinct from the RNA-null mutants (Fig. 5). An important future direction will be teasing apart protein-coding and non-coding functions for cncRNA loci using appropriate genome editing methods [150], [151]. This would require careful design of genomic lesions to specifically test phenotypic consequence of impaired ORF and ncRNA moieties. The identification and characterization of novel cncRNAs and the mechanisms by which they elicit their various functions, can provide new insights into gene regulation in the context of normal homeostasis and disease states.
