# Addendum

**DOI:** 10.1016/j.semcdb.2016.04.005

**Published:** 2016-05

Since the publication of the review by Sampath and colleagues entitled “cncRNAs: functional RNAs with protein coding and non-coding functions” (Semin Cell Dev Biol. 2015, 47–48: 40–51), another review article on dual function RNAs has been brought to our attention. We would like to make readers aware of the review by Ulveling et al., Biochimie, 2011, 93(4): 633–644.

